# Therapygenetics: Using genetic markers to predict response to psychological treatment for mood and anxiety disorders

**DOI:** 10.1186/2045-5380-3-4

**Published:** 2013-02-07

**Authors:** Kathryn J Lester, Thalia C Eley

**Affiliations:** 1 MRC Social, Genetic and Developmental Psychiatry Centre, Institute of Psychiatry, King’s College London, London, UK

**Keywords:** Genetics, Psychological therapy, Treatment response, Gene × environment interaction, Therapygenetics

## Abstract

Considerable variation is evident in response to psychological therapies for mood and anxiety disorders. Genetic factors alongside environmental variables and gene-environment interactions are implicated in the etiology of these disorders and it is plausible that these same factors may also be important in predicting individual differences in response to psychological treatment. In this article, we review the evidence that genetic variation influences psychological treatment outcomes with a primary focus on mood and anxiety disorders. Unlike most past work, which has considered prediction of response to pharmacotherapy, this article reviews recent work in the field of therapygenetics, namely the role of genes in predicting psychological treatment response. As this is a field in its infancy, methodological recommendations are made and opportunities for future research are identified.

## Review

Anxiety and depressive disorders are among the most common disorders with lifetime prevalence estimates of 28.8% for anxiety disorders and 20.8% for mood disorders [1]. They are some of the most disabling conditions and contribute a significant proportion of the worldwide burden of disease [2]. Psychotherapeutic approaches are recommended for the treatment of depression and all anxiety disorders [3], with the predominant approach being Cognitive Behaviour Therapy (CBT). In the UK, up to 10% of adults with anxiety or depression receive psychological treatments [4].

## Individual differences in psychological treatment response

CBT is not universally effective with response varying considerably between patients. While many people experience positive outcomes of therapy, approximately 35-45% of individuals retain significant impairments with a small number getting worse with treatment [5,6]. However, only limited research has investigated the source of this individual variation in psychological treatment response despite the potential of such work to guide treatment selection and improve outcome. Clinical and demographic characteristics have proven to be relatively weak or inconsistent predictors of who will respond to psychological treatment. Several authors have suggested that the most reliable predictors of response may come from the origins of the disorder, that is “cause should inform cure” [7,8].

There is an increasing interest in identifying biomarkers, which predict differential treatment response. These markers can be used to match particular groups of patients with treatments to which they are more likely to show a positive response from the outset in an approach termed “stratified medicine” [9]. Mood and anxiety disorders represent suitable therapeutic areas for stratified medicine to emerge. There is considerable disease variability probably reflecting multifactorial etiology or biologically distinct conditions not yet distinguishable on the basis of clinical presentations. There are also multiple relevant targets for therapeutic intervention and multiple treatment options with evidence of heterogeneity in response. These factors have been identified as necessary for the emergence of stratified medicine [9]. The causal pathways to mood and anxiety disorders involve a combination of genetic and environmental risk factors. Just as the genetic and environmental factors that contribute to the onset of these disorders differ across patients, so does the way that patients respond to the same treatment. Genetic (and environmental) factors are very likely to be involved in this differential response to treatment and thus represent plausible biomarkers.

In this article, we begin by briefly outlining the contribution of genetic, environmental and gene-environment interactions (G×E) to the etiology of mood and anxiety disorders. We then consider how the use of “therapeutic G×E” designs can be used to investigate whether genetic variants can be used to predict individual differences in response to positive environmental influences, namely psychological treatment interventions. We focus on the new field of “therapygenetics” [10] and review recent research that has investigated genetic predictors of individual differences in response to psychological therapy for mood and anxiety disorders. We conclude by outlining some important methodological considerations and potential developments for future research in this field.

## Genetic and environmental basis of mood and anxiety disorders

Mood and anxiety disorders are highly heterogeneous conditions. There are multiple causal pathways to these disorders involving various combinations of environmental and genetic risk factors, which alone are neither necessary nor sufficient for the disorder to develop [11,12]. Psychological illness tends to run in families strongly implicating genetics as a causal factor. This is confirmed by twin studies, which report that anxiety and mood disorders are moderately heritable [13,14]. While genetic risk factors do robustly predict onset, they are not a sufficient cause of these disorders. This is consistent with many monozygotic twins being concordant for a disorder, while others, despite their genetic similarity are discordant [15].

Environmental adversity, trauma and stress also often precede the onset of psychological illness implicating psychosocial factors in the causal pathway for mood and anxiety disorders. However, stress alone is not a sufficient cause: For example, only a minority (~20%) of those exposed to severe stressful life events (SLEs) go on to experience an episode of depression [16]. In others, mood and anxiety disorders emerge independent of recent stress [17]. This suggests considerable individual variation in the causal pathways to disease, which is mirrored in response to treatment: Not all patients will respond in the same way to the same treatment. Genetic and environmental factors, just as they are implicated in different etiological pathways to disorder may also determine individual variation in treatment response [18]. While this is a reasonable supposition, it is important to note that as yet there are no twin studies available that provide heritability estimates of response to psychological therapy [19].

## Gene-Environment interaction in mood and anxiety disorders

Genetic and environmental factors do not operate independently from each other and instead often co-occur in a causal pathway to disease within the context of gene-environment interaction. A G×E interaction occurs when a genetic and environmental factor participate in the same causal mechanism in the same individual [20]. For example, genetic variation may moderate the effects of the environment, placing some individuals at increased risk for disease, while making others resistant to the effects of negative or stressful environments.

The best-established and replicated G×Es for psychological disorders are the interaction between a functional variable number tandem repeat in the monoamine-oxidase-A gene (*MAOA*) and childhood maltreatment in the onset of antisocial and violent behaviour [21,22] and the interaction between the serotonin transporter gene functional length polymorphism (5*HTTLPR*) and childhood maltreatment in the etiology of depression [23,24]. Males carrying the low expression short alleles of the *MAOA*-u VNTR and with a history of childhood maltreatment have a greater risk for antisocial behaviour. However, the association between childhood maltreatment and antisocial behaviour is weak in those carrying the high expression long alleles [22]. Individuals with the low expression short (S) allele of the 5*HTTLPR* report significantly more depressive symptoms when exposed to recent SLEs or childhood maltreatment relative to those with the long (L) allele (see [25] but also [26,27] for contradictory findings). Similar findings have also been reported between the 5*HTTLPR*, childhood maltreatment and anxiety sensitivity, a risk factor for anxiety disorders [28]. Other studies have shown replicated findings between variants in the hypothalamic-pituitary-adrenal axis related genes (e.g. *FKBP5*, [29,30]; *CRHR1*, [31]; *NR3C1*, [32]) and neurotrophic genes (e.g. *BDNF* val66met [33,34]) and mood and anxiety disorders.

## Diathesis-Stress and differential susceptibility

Gene-environment interaction research has typically been couched in a diathesis-stress framework [35]. This framework proposes that some individuals due to a genetic vulnerability are disproportionately likely to be adversely impacted by an environmental stressor (e.g. SLEs, childhood maltreatment, insensitive parenting) compared to individuals carrying the “protective” genotype. The consequences being that those individuals carrying the “risk” genotype are more likely to develop a psychological illness when exposed to an adverse environment.

Some authors have argued that while diathesis-stress phenomena are an incontestable characteristic of human functioning and development, the situation is more nuanced than that suggested by this framework [36]. G×E studies have typically focused on a restricted range of environments, predominantly emphasizing adverse environments and the impact for negative psychological outcomes. Very few studies have measured positive environmental variables (aside from the absence of adversity) or considered the effects for adaptive functioning and outcomes (aside from the absence of dysfunction) [36].

The differential-susceptibility hypothesis [36-38] and biological sensitivity to context framework [39] argue that “vulnerability” genes may be a misnomer. Instead those individuals considered “vulnerable” by virtue of their genetic make-up and thus most adversely affected by negative environments may in fact benefit the most from enriched and supportive environments. In this way, individual differences in developmental plasticity and susceptibility to environmental influences may result in genetic influences that act in a “for better *and* for worse” manner [37]. Some individuals are more likely to be affected than others by *both* negative *and* positive environmental conditions. Data from quite a number of studies investigating a range of markers (e.g. *MAOA,* 5*HTTLPR, DRD2, DRD4, DAT1, TPH1, HT2RA*) have shown that individuals carrying the putative “risk allele” do show a for better and for worse pattern, functioning most poorly when exposed to negative environments but showing least problems when they encounter the absence of adversity or positive environments (see [36] for a review).

## Therapeutic GxE: genetic predictors of response to treatment

The interaction between a therapeutic intervention and genotype represents a special case of G×E [7]. Examining a positive experience within a gene-environment interaction framework is rare. The use of a therapeutic intervention, particularly a psychological intervention such as CBT is an unusually powerful G×E design. The “environment” is positive and predictable, thus allowing any potential moderating genetic effects to be investigated prospectively (unlike typical G×E designs where SLEs/maltreatment are unpredictable and commonly assessed retrospectively). Furthermore, it enables consideration of vulnerability markers that reflect differential susceptibility to the environment, be it positive or negative. By using “experimental manipulation” of G×E interactions, it is easier to rule out alternative explanations in terms of evocative or passive G×E correlations [40].

As an environmental factor, CBT and other psychological treatment interventions are likely to have a large and potentially long-standing impact. CBT is a learning-based intervention in which patients actively recall, reappraise and reconstruct their cognitions and behaviours. It leads to symptom reductions and changes in cognitions and behaviour (e.g. [41,42]) and there is some evidence that CBT also alters neural function with the changes consistent with the reduction in symptoms seen following treatment (see [43]). There is preliminary evidence that CBT may also stimulate structural brain changes [44]. While not yet investigated in the context of psychological treatments, it is plausible that the mechanisms of action for CBT may also include changes in gene expression and neurogenesis as has been observed for antidepressants [45,46].

Genetic predictors of response to pharmacological treatments (pharmacogenetics) for mood and anxiety disorders have been widely investigated using both candidate gene and genome-wide (GWAS) methodologies. Some evidence suggests that variants implicated in the pathogenesis of the disorder may also predict response to antidepressants [47]. For example, response to selective serotonin reuptake inhibitors (SSRIs) but not tricyclic antidepressants is predicted by the presence of one or more copies of the high expression L allele of the 5*HTTLPR*[48]. However, this association has failed to replicate in several large studies and overall a meta-analysis reported a nonsignificant effect [49]. Genome wide studies have also identified plausible candidate genes [50-52]. Currently, the strength of findings from pharmacogenetics studies and the lack of replication mean that genetic associations are not yet sufficiently strong or reliable to guide stratified treatment selection [53].

Very few studies have investigated genetic predictors of individual differences in response to psychological or psychosocial interventions, a field we recently termed “therapygenetics” [10]. Early work in this field focused on family interventions and outcomes in child samples e.g. [40,54] and produced findings consistent with a differential susceptibility framework in which the same genetic variant operated in a for better and for worse manner [55]. More recent work has investigated genetic predictors of response to psychological treatment interventions, with the majority of studies to date focusing on mood and anxiety disorders.

## Therapygenetics studies in mood and anxiety disorders

### Literature search methodology

Key word searches were performed in Web of Knowledge using the following terms: “therapygenetics”; “genetics” and “cognitive behavior therapy” and “response”; “genes” and “psychotherapy”. Search results were reviewed for potentially relevant empirical papers and 13 relevant papers were identified, which had investigated genetic predictors of response to a psychological intervention. Of those, 10 had mood or anxiety as the main disorder of interest and the remainder focused on an alternative psychiatric disorder and are not reviewed here. Four studies on mood and anxiety disorders were not yet indexed in Web of Knowledge or had only been presented in (non-indexed) conference papers but were made available to us for inclusion by the authors. Six additional studies were identified by inspection of reference lists of included studies and relevant review articles. However, as these focused on other psychiatric disorders or response to family-based interventions with children and adolescents, these studies are not considered further.

### Mood and anxiety disorders

Table 1 summarises the therapygenetics studies published to date, where clinically diagnosed anxiety or depression are the disorder phenotype of interest. The field is in its infancy and this is reflected in the small, highly heterogeneous and preliminary nature of the existing literature. In particular, sample sizes have ranged from very small pilot studies (e.g. *N* = 45, [56]) to somewhat larger investigations (e.g. *N* = 374, [57]). However, modest sample sizes mean the vast majority of studies are underpowered to detect what we expect to be small genetic effects. Presently, this limits our ability to draw strong conclusions regarding the predictive ability of genetic variants for psychological treatment response.

**Table 1:** Summary of studies investigating associations between genetic variation and response to psychological therapy in mood and anxiety disorders

**Authors**	**Diagnosis**	**Sample**	**Treatment**	**Results**
**5HTTLPR/rs25531**				
Wang et al. [58]	PTSD	*N* = 35	12wk prolonged exposure therapy or 12 wk Escitalopram (N = 20)	No significant association with treatment response in exposure therapy group
Bryant et al. [56]	PTSD	*N* = 45, Caucasian,	8 × 90 min weekly individual CBT	No significant association at post-treatment. At 6mth follow-up, higher % of *S*^*i*^ allele than *L* allele carriers met PTSD criteria and had significantly higher symptom scores
		*M*_age_ = ~ 43 yrs, 33% female		
Lonsdorf et al. [59]	PD ± AG	*N* = 69, Caucasian,	10 × 2 hr weekly group (*N* = 38) or internet-delivered (*N* = 31) CBT	No significant association with treatment response
		*M*_age_ = ~ 35 yrs, 62% female		
Furmark et al. [60]	SAD	*N* = 204, *M*_age_ = 38 yrs,	9 wk internet delivered CBT or waitlist control	No significant association with treatment response
		60% female		
Kohen et al. [61]	DEP (post- stroke)	*N* = 61, mixed ethnicity	9 × positive problem solving plus antidepressant vs. usual care plus antidepressant	*SS* and *SL* carriers had a significantly greater mean percentage reduction in depression ratings and more likely to be in remission at 9-week follow-up than those in the control group
Sakolsky et al. [62]	SEP; GAD; SAD	*N* = 211, Caucasian,	Sertraline, 14 sessions of CBT, combination therapy or 12 wk placebo	No significant association with treatment response
		7-17 yrs,		
Eley et al. [10]	ANX	*N* = 359, Caucasian,	10-12 session group or individual CBT or guided self-help	No significant association at post treatment. At follow-up, higher % of *SS* genotype carriers free of anxiety diagnoses than *SL/LL* genotype carriers. *SS* genotype carriers had significantly greater reduction in symptom severity scores
		*M*_age_ = 9.44 yrs, 49% female		
Hedman et al. [63]	SAD	*N* = 126, 98% Caucasian, *M*_age_ = ~ 35 yrs, 36% female	15 × 2.5 hr weekly group (*N* = 62) or internet-delivered (*N* = 64) CBT	No significant association with treatment response
Bockting et al. [64]	Recurrent DEP	*N* = 180, Caucasian,	Brief CBT vs. treatment as usual	No significant association with time to recurrence
		*M*_age_ = 45 yrs, 74% female		
**STin2 VNTR**				
Kohen et al. [61]	DEP (post- stroke)	*N* = 64, mixed ethnicity	9 × positive problem solving plus antidepressant vs. usual care plus antidepressant	9/12 and 12/12 genotype carriers in intervention group had a significantly greater mean percentage reduction in depression scores and greater likelihood of remission at 9 wk follow-up than controls
Sakolsky et al. [62]	SEP; GAD; SAD	*N* = 211, Caucasian,	Sertraline, 14 sessions of CBT, combination therapy or 12 wk placebo	At 12-week assessment *STin2* 12-copy variant carriers showed significantly greater improvement*.*
		7-17 yrs		
**HTR2A rs7997012**				
Kotte et al. [65]	DEP	*N =* 58, 100% male	16-wk group CBT	G allele predicted significantly larger reduction in BDI scores across treatment compared to A allele carriers
**TPH2 G-703T**				
Furmark et al. [60]	SAD	*N* = 204, *M*_age_ = 38 yrs,	9 wk internet delivered CBT or waitlist control	In the CBT group, a better treatment response was observed in *TPH2* GG homozygotes relative to T allele carriers
		60% female		
**MAOA-u VNTR**				
Reif et al. [66]	PD + AG	*N* = 288, Caucasian	12 × twice weekly CBT	Carriers of the long, higher activity allele^ii^ had significantly worse treatment outcome, elevated heart rate, greater fear and panic attacks during a behavioral avoidance task and failure to habituate during repetitive exposure
**COMT val158met**				
Lonsdorf et al. [59]	PD ± AG	*N* = 69, Caucasian,	10 × 2 hr weekly group (*N* = 38) or internet-delivered (*N* = 31) CBT	No significant effect of COMTval158met genotype on change in anxiety or depression scores across cognitive modules (weeks 1-3). met/met genotype carriers had significantly smaller reduction in anxiety scores across exposure modules (weeks 4-9) compared to val-carriers
		*M*_age_ = ~ 35 yrs, 62% female		
Hedman et al. [63]	SAD	*N* = 126, 98% Caucasian, *M*_age_ = ~ 35 yrs, 36% female	15 × 2.5 hr weekly group (*N* = 62) or internet-delivered (*N* = 64) CBT	No significant association with treatment response
**NGF rs6330**				
Lester et al. [57]	ANX	*N* =374, Caucasian,	10-12 session group or individual CBT or guided self-help	No significant association with treatment response at post treatment. At follow-up, children with one or more copies of T allele of *NGF* rs6330 were significantly more likely to be free of anxiety diagnosis
		*M*_age_ = 9.46 yrs, 49% female		
**BDNF val66met; rs7934165; rs1519480; rs11030104**				
Sakolsky et al. [67]	SEP; GAD; SAD	*N* = 211, Caucasian,	Sertraline, 14 sessions of CBT, combination therapy or 12 wk placebo	No significant association with treatment response
		7-17 yrs		
Lester et al. [57]	ANX	*N* = 374, Caucasian,	10-12 session group or individual CBT or guided self-help	No significant association with treatment response
		*M*_age_ = 9.46 yrs, 49% female		
Hedman et al. [63]	SAD	*N* = 126, 98% Caucasian, *M*_age_ = ~ 35 yrs, 36% female	15 × 2.5 hr weekly group (*N* = 62) or internet-delivered (*N* = 64) CBT	No significant association with treatment response
Fullana et al. [68]	OCD	*N* = 106, Caucasian,	20 × 45 min weekly exposure based CBT plus SSRI	Met allele carriers significantly less likely to respond to treatment than non-met allele carriers. Genotype predicted response only and not change in severity scores
		*M*_age_ = 33 yrs, 50% female		
**GRIN2B rs1019385**				
Sakolsky et al. [67]	SEP; GAD; SAD	*N* = 211, Caucasian,	Sertraline, 14 sessions of CBT, combination therapy or 12 wk placebo	No significant association with treatment response
		7-17 yrs		
**GRIK4 rs1954787**				
Sakolsky et al. [67]	SEP; GAD; SAD	*N* = 211, Caucasian,	Sertraline, 14 sessions of CBT, combination therapy or 12 wk placebo	Significant association with treatment response at 12 week assessment
		7-17 yrs		

*PTSD* = post-traumatic stress disorder; *PD ±AG* = panic disorder with or without agoraphobia; *SAD* = social anxiety disorder; *DEP* = depression; *SEP* = separation anxiety disorder, *GAD* = generalized anxiety disorder; *ANX* = all anxiety disorders; *OCD* = obsessive compulsive disorder.

^I^*S* allele group defined as S_A_S_A_, S_A_L_A_, S_A_L_G_, S_G_L_G_, L_A_L_G_, L_G_L_G_ and *L* allele group defined as L_A_L_A._^ii^ Long, high activity risk allele group defined as 3.5, 4 and 5 copy repeat allele males, and 2/4, 3/4, 3.5/4, 4/4, and 4/5 copy repeat allele females. Short, low activity allele group defined as 2 or 3 copy repeat allele males and 2/2, 2/3 and 3/3 females.

Studies thus far have relied on a candidate gene approach, testing the effect of a single marker or limited number of genetic polymorphisms within a gene on psychological treatment response. The most widely assessed polymorphism has been the 5*HTTLPR*. In the following section, we briefly outline the rationale for each genetic marker being a plausible candidate for involvement in psychological treatment response. We then review the findings of therapygenetics studies to date, grouped by the markers that have been considered. In light of the infancy of the therapygenetics field, we then draw attention to several methodological issues for consideration in future research.

#### Serotonin transporter linked polymorphic region (5HTTLPR)

Serotonergic neurotransmission is associated with anxiety and mood disorders and is implicated in the treatment of both types of disorder. By far the most widely studied polymorphism has been the 5*HTTLPR*, which is a 43 bp insertion deletion functional polymorphism in the 5’ promoter region of the *SLC6A4* gene, resulting in short and long alleles, which differ in 5-HTT expression and function. The short allele variant is associated with approximately 50% less 5-HTT expression and function leading to higher concentrations of serotonin in the synaptic cleft [69]. The 5*HTTLPR* is often studied in conjunction with the A/G single nucleotide polymorphism (SNP) rs25531, which has been shown to differentially impact the L allele function. The minor G allele is commonly in phase with the long allele of the 5*HTTLPR* with the L_G_ allele reported to have transcriptional activity similar to the S allele [70]. Participants are often classified on the basis of presumed efficacy of serotonergic neurotransmission into low (SS, SL_G_, or L_G_L_G_)_,_ intermediate (SL_A_ or L_A_L_G_) and high (L_A_L_A_).

The evidence for an association between the 5*HTTLPR* and mood and anxiety disorders has been somewhat mixed. The low expression allele has most often been associated with greater affective and anxiety-related traits (e.g. [71-73]) and related intermediate phenotypes (e.g. attentional bias to emotional stimuli [74]; fear conditioning [75,76]; amygdala reactivity [77]). Consistent with the differential susceptibility hypothesis, a recent meta-analysis showed that the low expression allele was associated with poorer outcomes following stress ([25], but see also [27] for nonsignificant effects) but also appears to be associated with better outcomes under low stress or positive environments [36]. This led to the hypothesis that the low expression S allele would be associated with enhanced response to psychological therapies [10].

Nine studies have tested for an association between the 5*HTTLPR* and response to psychological therapy with three studies reporting evidence of a significant association. With respect to the five negative studies, there was no significant association between the 5*HTTLPR* and relapse rates following a course of CBT in remitted recurrently depressed adults (*N* = 180, [64]); improvement in symptoms in response to internet based CBT for socially anxious adults (*N* = 204, [60]; *N* = 126, [63]); improvement in anxiety symptom scores in response to exposure based CBT in panic disorder patients (*N* = 69, [59]) and improvement in symptoms in response to multimodal treatment in child anxiety disorders (*N* = 211, [62]). All of these studies have small samples and are therefore underpowered to detect what are very likely to be small genetic effect sizes.

With regard to positive studies, anxiety-disordered children (*N* = 359) carrying the low expression (SS) genotype were significantly more likely than the intermediate (SL)/high (LL) expression genotype carriers to be free of their anxiety disorder diagnosis (78.4% vs. 58.4%) and to have a larger reduction in symptom severity scores after a course of CBT or guided self-help [10]. In the second positive study (*N* = 61), the low expression (SS) genotype was associated with a greater percentage change in depression scores in clinically depressed older adults after ischemic stroke and in response to a brief problem solving plus antidepressant intervention [61]. These findings appear consistent with a differential susceptibility explanation in which the same genetic variant, the low expression S allele, which in prior studies has been associated with increased risk for anxiety and depression, is also associated with the most improvement in response to a positive psychological treatment environment.

In contrast to the direction of effect observed in the two previous studies [10,61], a third study found that the low expression genotype was associated with more severe PTSD symptoms and therefore fewer treatment gains at 6-months in patients undergoing exposure-based CBT for PTSD (*N* = 45; [56]). In this study, the authors suggest that the low expression allele may limit the capacity to gain control over the anxiety elicited through CBT as a consequence of greater amygdala reactivity, stronger fear conditioning and deficits in extinction learning [56]. These findings are also consistent with past (albeit mixed) research reporting an association between the low expression genotype and poorer response to SSRIs [78]. In a further study, PTSD patients received a 12-week trial of citalopram (*N* = 20) or prolonged exposure therapy (*N* = 15, [58]). The low expression SS genotype was again associated with a poorer treatment response in both treatment groups with the effect only attaining statistical significance in the pharmacotherapy group [58]. The contradictory findings observed for 5*HTTLPR* may reflect not only small sample sizes and varying clinical phenotypes, but also the role of medication. Further adequately powered studies are required to clarify the association between 5*HTTLPR* and response to psychological treatment.

#### Serotonin transporter intron 2 variable number tandem repeat (STin2 VNTR)

The *STin2* VNTR of the 5-*HTT* gene has multiple repeated copies of a 16-17bp element [79]. Nine (STin2.9), ten (STin2.10) and 12 (STin2.12) repeat alleles have been identified. Different copy number variations of the VNTR have been shown to have differential regulatory effects on transcription [80,81]. The short variant (9 or 10 repeats) is associated with lower levels of 5-HTT mRNA and has been linked with unipolar depression [82] and anxiety [83]. However, others have reported an association between STin2.12 alleles and neuroticism [84] and anxiety disorders [85].

Variation in *STin2* has been examined in two therapygenetics studies. In the first, post-stroke depressed patients with 9/12 or 12/12 repeat alleles showed a larger percentage reduction in depression scores to a problem-solving intervention (*N* = 64, [61]). Similar significant associations with treatment response were reported in a second study of children with a diagnosis of separation anxiety disorder, generalized anxiety disorder or social phobia receiving either sertraline medication, CBT or a combination therapy (*N* = 211, [62]). Significantly greater improvement defined by Clinical Global Impression-Improvement scores of very much or much improved at post-treatment was observed in carriers of the 12-copy variant after controlling for age and treatment condition. The findings from these two studies are in contrast to those reported in some studies for response to SSRIs. For example, poorer remission rates from major depression were reported in carriers of the 12/12 genotype in the Sequenced Treatment Alternatives to Relieve Depression study (STAR*D [86]).

#### 5-hydroxytryptamine (serotonin) receptor 2A gene (HTR2A)

The *HTR2A* gene encodes for 5-hydroxytryptamine 2A receptors, which have been implicated in animal and human models of depression and are thought to play an important role in antidepressant drug action [87]. Several polymorphisms in this gene have been studied in relation to mood and anxiety disorders although associations have tended to be small and findings have been mixed [88,89]. The most widely studied polymorphisms have been A-1438G (rs6311); C102T (rs6313) and His452Tyr (rs6314). There is some evidence that genetic variants in the *HTR2A* gene may predict differences in response to SSRI treatment [87,90]. For example, a further SNP, rs7997012, located in intron 2 of the *HTR2A* gene has been associated with treatment response to antidepressants in the STAR*D sample in both a discovery and replication sample. Being homozygote for the A allele was associated with a 16-18% reduction in risk of non-response compared to GG homozygote carriers [87,90].

A single therapygenetics study has tested for an association between the *HTR2A* rs7997012 polymorphism and response to a 16-week course of CBT in a sample of 58 male veterans experiencing unipolar depression [65]. In contrast to the STAR*D studies investigating associations with antidepressant response, preliminary findings showed that carriers of the G allele had a significantly larger reduction in depression symptom scores in response to CBT treatment than AA homozygotes.

#### Tryptophan hydroxylase 2 gene (TPH2)

The neuronal isoform of the *TPH2* gene is expressed exclusively within the nervous system and codes for the rate-limiting enzyme for 5-HT biosynthesis in midbrain and serotonergic neurons [91]. *TPH2* has previously been associated with depression [92] and anxiety disorders [93], although findings have not always replicated. The SNP G-703T (rs4570625) is located in the promoter region of the *TPH2* gene. It is thought to play a functional role in regulating 5HT signaling and has a significant effect on gene expression with the T allele associated with reduced transcriptional activity [94] and increased amygdala reactivity to emotional stimuli [95,96].

A single therapygenetics study has tested for an association between the *TPH2* G-703T polymorphism and response to a 9-week internet-based CBT intervention for social anxiety disorder (*N* = 204, [60]). A significantly greater improvement in self-report social anxiety symptom scores was observed in GG homozygote individuals compared to T allele carriers at post-treatment. Interestingly, in an earlier paper the GG genotype significantly predicted greater improvement in social anxiety symptoms in response to a placebo drug condition [95]. Statistical analyses were consistent with the improved treatment response in GG genotype carriers being mediated by its effect on attenuating amygdala activity. This suggests a possible biological mechanism linking genetically controlled serotonergic modulation of amygdala reactivity and treatment response. One might anticipate a similar mediation mechanism linking *TPH2* polymorphism and CBT response given that placebo treatments are thought to act on essentially the same neural pathways as those influenced by active treatments [97,98]. This association with placebo response also raises the interesting possibility that the G-703T GG genotype may not be specifically associated with therapeutic responsiveness but rather a more general tendency to recover irrespective of treatment. This can be best clarified further by the inclusion of a no treatment control group alongside active and placebo treatment conditions.

#### Monoamine oxidase-A variable number tandem repeat (MAOA-u VNTR)

*MAOA* is a logical candidate gene for involvement in psychiatric disorders due to its role in metabolizing serotonin and norepinephrine [99]. A 30 bp VNTR polymorphism called *MAOA*-uVNTR in the promoter region of the gene has recently been studied with regard to psychological treatment response. This VNTR has a long allele (3.5, 4 and 5 repeats) and short allele (2 and 3 repeats). The long alleles demonstrate increased transcriptional activity [100,101] with levels of 5-hydrocyindoleacetic acid (5-HIAA), the main metabolite of serotonin influenced by this polymorphism [102]. The *MAOA*-uVNTR is often referred to as the warrior-worrier gene [103] due to short-allele repeats being associated with impulsive-aggressive behavior in males [21,104,105]. Long repeat alleles have been associated with panic disorder in females [100,106] and heightened sensitivity to aversive experiences [107].

Only one therapygenetics study has explored variation in the *MAOA*-u VNTR [66]. In a sample of 288 patients diagnosed with panic disorder with agoraphobia and receiving exposure-based CBT, carriers of the long repeat higher activity risk allele had a significantly worse treatment response (46% responders) than homozygote carriers of the short repeat allele (67% responders) [66]. In response to a behavioural avoidance test involving entering a small dark chamber, L allele carriers experienced significantly higher heart-rate during anticipation of the task, as well as during exposure and recovery phases, more intense anxiety during exposure, a greater frequency of panic attacks and a failure to habituate. The authors argue that the poorer outcome seen in L allele carriers is consistent with a possible effect of *MAOA* on the efficiency of extinction learning via decreased noradrenergic availability [66]. This would be consistent with evidence that adrenergic enhancers facilitate exposure based CBT for anxiety disorders (e.g. [108]).

#### Catechol-O-methyltransferase gene (COMT)

Catechol-*O*-methyltransferase is a methylation enzyme encoded by the *COMT* gene that acts to degrade monoaminergic neurotransmitters (e.g. dopamine, epinephrine, norepinephrine) [109]. An A/G SNP, *COMT*val158met (rs4680) codes for a valine (val) to methionine (met) exchange at position 158. The val allele is associated with a three to four-fold increase in functional activity of the methylation enzyme compared to the met allele, which impacts on the degradation of dopamine and leads to an excess of synaptic dopamine in met carriers [110,111]. Associations have been reported between the *COMT*val158met polymorphism and fear extinction processes (for a review see [19]) and psychopathology, although the direction of effects has not proven entirely consistent. While COMT genotype was not found to be associated with indices of fear conditioning [75,112], there was some evidence that the met allele was associated with stronger and extinction-resistant fear memories using a 24-hour delayed extinction paradigm [75]. However, there was no significant association with extinction learning in a further study using an immediate extinction procedure [112]. The val/val genotype has also been associated with enhanced fear reacquisition under certain experimental conditions [76]. With regard to psychopathology, the met allele has also been associated with severity of PTSD [113], and phobic avoidance and panic attacks in adolescent females [114]. However, the val allele has also been associated with panic disorder [115].

Two therapygenetics studies have tested for an association between the *COMT* gene and psychological treatment response. Building on experimental studies of fear conditioning and extinction, the *COMT*val158met was considered for association with efficacy of group or internet-delivered exposure-based CBT in a sample of 69 panic disorder patients [59]. No significant association with genotype was observed for change in depression symptom scores across treatment or for change in anxiety symptom scores across the cognitive treatment modules, which occurred early in treatment. However, met/met genotype carriers reported a significantly smaller reduction in anxiety symptoms across the exposure treatment modules compared to val carriers. This effect remained significant even after controlling for demographic and clinical variables and 5*HTTLPR*/rs25531 genotype. The authors suggest that impaired top-down cognitive control over emotional reactions and a related failure to extinguish fear reactions may represent one pathway via which the met/met group gains less benefit from psychological therapy [59]. In the only other therapygenetics study to investigate the *COMT*val158met, no significant association with response to group or internet delivered CBT was observed in a sample of 126 patients diagnosed with social anxiety disorder [63]. It is possible that differences in disorder phenotype (e.g. PTSD vs. SAD) may account for the discrepancy in findings or that *COMT* genotype may be of greater relevance when treatment modality is predominantly exposure based rather than cognitive based. Further research is needed to test these hypotheses.

#### Nerve growth factor (NGF)

A number of therapygenetics studies have focused on candidate genes that are purportedly implicated in the pathophysiology of learning with the rationale being that CBT, and exposure based CBT especially rely on the basic principles of extinction learning [116]. *NGF* is a neurotrophic gene that is thought to have a key role in neuronal survival, activity-dependent neuroplasticity and learning, is implicated in the orchestration of response to stress and is widely expressed in limbic areas of the central nervous system involved in mood and cognition [117]. In *NGF*, rs6330 has been investigated as a predictor of response to psychological therapy. This non-synonymous SNP produces an alanine to valine substitution at amino acid position 35 and is thought to have a possible functional role on intracellular processing and secretion of *NGF*[118]. Allelic variation at this locus has shown associations with anxiety-related traits and affective disorders [119,120].

Variation in *NGF* rs6330 has been examined in a single therapygenetics study to date. Likelihood of remission increased with each extra T allele of the *NGF* rs6330 marker in a sample of anxiety-disordered children after a course of CBT or guided self-help (*N* = 384, [57]). Fifty-three percent of CC genotype carriers were free of their primary anxiety diagnosis compared to 63.5% and 76.7% for CT and TT genotypes respectively. However, the effect of genotype was observed only at follow-up assessment and not immediately post-treatment. One possibility is that the *NGF* T allele may influence capacity for continued benefit from CBT. The authors suggest that children with the T allele may show subtle differences in neurotrophic signalling that influence the extent to which environmental influences, in this instance therapeutic interventions bring about neuroplastic modifications, which in turn modulate mood [57].

#### Brain-derived neurotrophic factor gene (BDNF)

*BDNF*, like *NGF* is also a neurotrophic gene. *BDNF* secretion is activity dependent with decreases associated with stress, mood disorders and anxiety-related behaviour and increases with antidepressant medication [121]. The most frequently studied polymorphism in the *BDNF* gene has been the functional val66met (rs6265) polymorphism. In val66met, the more common G allele encodes for valine (val) with the A allele encoding for methionine (met). The met allele is associated with diminished activity-dependent secretion of *BDNF*[122,123], abnormalities in brain structure and function in limbic regions [124-126]. The val66met polymorphism has also been studied in conjunction with associative fear learning, fear generalisation and extinction processes, albeit with mixed findings (for a review see [19]).

Four therapygenetics studies have tested for an association between the *BDNF* gene and psychological treatment response. The most frequently studied polymorphism has been the val66met (rs6265) polymorphism, however other polymorphisms within this gene have also received attention (rs7934165, rs1519480, rs11030104). Three studies failed to find any association between *BDNF* variants and response to psychotherapy for child anxiety disorders (*N* = 374, [57]); response to multimodal treatment for child anxiety disorders (*N* = 211, [67]) or adult social anxiety disorder treated with group or internet-based CBT (*N* = 126, [63]). However, a single study reported an association between the val66met polymorphism and response to exposure-based CBT in a sample of 106 OCD patients who had previously shown partial or non-response to a 12-week pharmacological trial [68]. Thirty-six percent of met allele carriers compared to 60% of val carriers responded to treatment defined as a 35% or greater decrease in Yale-Brown Obsessive Compulsive Scale scores. Of note, all participants also received concurrent SSRI treatment in this study and the treatment modality was predominantly exposure based rather than cognitive based. While warranting further investigation, these methodological differences may in part account for the discrepancy with other studies.

#### Glutamate receptor, ionotropic N-methyl D-asparate 2B gene (GRIN2B)

The *GRIN2B* gene encodes the NR2 subunit of the *N*-methyl-D-aspartate (NMDA) glutamate receptors, which are important for fear acquisition and fear consolidation [127] and learning and memory more broadly [128]. The SNP, rs1019385 located in NMDA 2 beta subunit is a promoter region variant in which the G allele leads to reduced transcriptional activity which in turn may affect glutamate neurotransmission [129]. *GRIN2B* has been associated with childhood OCD [130], and related biological traits including reduced anterior cingulate glutamatergic concentration [131]. However, in the only therapygenetics study to type this marker there was no significant association between *GRIN2B* rs1019385 and response to CBT, pharmacotherapy or combined treatment defined by improvement scores in a sample of children with anxiety diagnoses (*N* = 213, [67]). Note, however, that no children with a primary OCD diagnosis were included in this sample.

#### Glutamate receptor, ionotropic kainite 4 gene (GRIK4)

The *GRIK4* gene encodes for a kainate preferring ionotropic glutamate receptor subtype involved in modulating neurotransmitter release and excitatory neurotransmission. The SNP, rs1954787 located in the 3’ end of the first intron is not thought to alter protein sequence but may have functional relevance via regulation of gene expression [90]. A recent study reported an association between the C allele of this marker and improved treatment response to antidepressants in the STAR*D cohort [90] but this effect did not replicate in the Munich Antidepressant Response Signature sample after correction for the number of SNPs tested [132]. This marker has been investigated in one therapygenetics study to date. A significant association between the rs1954787 SNP and treatment response defined as a rating of very much or much improved on the Clinician Global Impression – Improvement scale was observed after controlling for age, treatment condition and other genetic polymorphisms investigated in a sample of children with anxiety diagnoses (*N* = 213, [67]). However, it is unclear whether *GRIK4* genotype interacts with treatment type as some children received medication, others CBT and some a combined therapy.

#### Methodological considerations

A small number of studies have provided preliminary but provocative evidence in support of an association between genetic polymorphisms and response to psychological therapy. Independent replication of these early findings in adequately powered samples should be a priority. Several other studies have failed to find any evidence for a significant association or have produced conflicting results. In this section we outline some of the methodological issues and directions for future research, which we believe should be considered in future therapygenetics research.

### Sample characteristics

Therapygenetics studies have been conducted with child, adult and older adult samples. At this stage, no particular pattern has emerged to suggest that genetic predictors of psychological treatment response may be more or less likely to be observed in younger versus older samples. However, developmental factors are an important consideration. Twin studies have identified that genetic and environmental influences on depression change across adolescence (e.g. [133]). It is therefore plausible that new or different genetic effects or environmental experiences may emerge at different points across development and which may be instrumental in determining psychological treatment response. Other participant characteristics, for example, gender, socio-economic status, educational levels and motivation may also interact with genetic factors to influence psychological treatment response. Consideration of the ethnic background of the sample is also very important for genetic association studies. Thus far, most therapygenetics studies have employed samples with predominantly Caucasian ancestry. The use of ancestrally homogenous samples or appropriate statistical techniques (e.g. principal components approaches) to control for ancestral heterogeneity is very important as by doing so any risk associated with hidden population substructures leading to spurious results is attenuated.

### Treatment characteristics

In therapygenetic studies conducted to date, the psychological treatment variable has not been consistent. Multiple CBT treatment formats have been used, which differ with respect to aspects such as whether the treatment was delivered face to face or over the internet, in an individual or group format, predominantly cognitive or exposure based in focus, the “dose” of treatment delivered and whether parents were involved in the treatment process. CBT is also a multimodal treatment combining a variety of cognitive and behavioural components. Some authors have suggested that genetic variants might have greater predictive power for more focused psychological treatments [134]. Inspection of the present findings does not reveal any particularly strong and compelling pattern of effects. Ultimately, if genetic predictors are to be useful clinically then any predictive effects would need to rise above this noise.

Several of the studies reviewed also included participants who received a combined treatment comprising both psychological therapy and medication (e.g. [61-63,67,68]). This raises an interpretive challenge as it becomes difficult to disentangle which component of treatment is associated with any genetic predictive effects. Combined treatment approaches may also mask and reduce any potential predictive effects of genetic variants for response to psychological therapy.

The majority of studies to date have not included a no-treatment control group. The advantage of such a group would be to distinguish between association with recovery time per se and recovery following psychological treatment. Where genes predict response in the active treatment but not no-treatment control group it would be possible to conclude with greater certainty that any genetic effects observed were specifically predictive of therapeutic response and not just a general tendency toward improvement across time. The use of wait list control groups are sufficient to test this hypothesis with respect to genetic associations with response immediately post-treatment. However, the withholding of a broadly efficacious intervention for the extended period of time needed to test this hypothesis with respect to genetic associations at a follow-up assessment cannot be easily justified for ethical reasons.

### Disorder characteristics

Accurate and reliable characterisation of the disorder phenotype will continue to be important. We advocate the use of semi-structured diagnostic interviews and clinician scored rating scales as these provide an objective measure of diagnosis, functioning and clinical improvement. Self-report scales have a number of methodological challenges (e.g. biases in reporting), which may potentially lead to an inaccurate reporting of symptoms [135]. How treatment response is defined is also important. Some studies have defined positive treatment response in terms of remission of the primary diagnosis, others in terms of cut-offs based on percentage reduction in symptom severity scores, some have looked at raw symptom scores and finally a small number of studies have investigated related phenotypes including time to recurrence. As the field progresses it will be important for some consistency to emerge with respect to defining response. This is particularly important because to amass the large sample sizes necessary to achieve sufficient power to reliably detect genetic effects will require collaboration amongst research teams. This inevitably brings with it problems in ensuring that the treatment and clinical measures are sufficiently similar to permit the combination of samples. The selection of appropriate measures should perhaps be guided by what is most clinically meaningful. Our recommendations would be remission of the primary diagnosis of interest and ratings of improvement using the Clinician Global Impression Improvement scale.

### Molecular genetic and statistical considerations

Studies so far have focused on a small number of predominantly functional candidate markers in a limited number of genes. Immediate efforts should be directed toward attempting replication of the most promising findings published to date using adequately powered samples. In selecting targets for future candidate gene studies it has been suggested that a focus on variants implicated in the etiology of the disorder may prove to be the most robust predictors of response [7,8]. However, other genetic variants not directly implicated in etiology are also likely to prove important. With increasing sample sizes we anticipate that studies may move away from candidate gene approaches to hypothesis free genome wide association designs in conjunction with genome wide complex-trait analysis methods [136]. However, perhaps as a caution it is noteworthy that GWAS studies have yet to report replicated SNPs associated with antidepressant response for depression (see [50-52]). Integrating genotype information with epigenetic and gene expression data and with neurophysiological, psychological and behavioural measures may prove important in terms of identifying possible biological and psychological mechanisms that mediate the effect of genetic variants on psychological treatment response.

Given that psychological treatment response, like anxiety and depressive disorders is a complex trait, it is unlikely that any single gene in isolation will explain a sufficiently large amount of the variance in response to psychotherapy to be clinically meaningful in its own right (although with a sufficient sample size even very weak predictive effects may obtain statistical significance). Sample sizes to date have been relatively small which will have limited power to observe statistically significant effects. Even the largest study to date only had a total sample size of 374 [57]. It has been suggested that effects explaining as little as 1% of the variance should be considered important [137] - the *NGF* rs6330 polymorphism accounted for 2.3% of the variance in treatment outcome. This is a relatively encouraging effect size given that gene-environment interactions are often modest in size and represent a substantial methodological challenge in terms of requiring relatively large samples to obtain sufficient statistical power and careful and accurate measurement of treatment outcome. However, it is widely reported that new association findings tend to overestimate the true size of the effect due to a phenomenon known as the winner’s curse [138]. This overestimation in initial studies can cause replication studies to fail due to them being underpowered. However, independent replication studies and at a later stage, meta-analyses are essential if the field of therapygenetics is to progress and yield potentially clinically useful applications.

### Future directions

At present, genetic predictors do not have sufficient predictive power to warrant their use as a clinical biomarker. Moving toward analytic methods that aggregate across multiple polymorphisms and/or genes into a single parameter in combination with machine learning methods may be the most powerful means of achieving clinically significant prediction and building toward a stratified medicine approach for mood and anxiety disorders [134,139]. Combining genetic information with clinical and demographic predictors [140] or perhaps with neuroimaging biomarkers [141] within a single algorithm may also prove to be a particularly powerful approach. More broadly, the inclusion of clinical covariates within models designed to test association between specific SNPs and psychological treatment outcome is important. Clinical predictors such as baseline symptom severity, the presence of comorbid disorders and familial history of illness are themselves likely to be under genetic influence. It is therefore important to show that any predictive effect of a genetic variant exists above and beyond the effect of any significant clinical predictors.

For an individual patient, the key clinical decision is not whether to treat or not but instead which treatment to select to maximize the chance of recovery [7]. In these circumstances, using genetic markers to predict response to a particular treatment is of limited clinical utility. Instead, as a medium term aim, therapygenetics studies need to move away from predicting response to a single psychological treatment and instead should aim to identify differential predictors of response to alternative treatment modalities. For example being able to estimate on the basis of genetic information, the relative likelihood of response to pharmacotherapy versus psychotherapy would be of considerably greater clinical value than identifying that a particular patient is more or less likely to respond positively to SSRIs. However, it may not be realistic to expect to find a genetic variant associated with response to pharmacological treatment per se given the different biological pathways via which different classes of drugs exert their influence. One way to progress might be to try and identify genetic variants that differentially predict response to a specific psychological therapy e.g. CBT compared to response to a specific class of drugs e.g. SSRIs or MAOIs. Unreplicated research suggests an interesting potential double dissociation between 5*HTTLPR* genotype and response to SSRIs and CBT treatment modalities. In adult depression, response to SSRIs has been shown to better in LL carriers of the 5*HTTLPR*[47], while some therapygenetics studies have observed better response to CBT in SS carriers [10,61]. However, it is important to note that several studies failed to find any association between 5*HTTLPR* and response to psychological therapy [58-60,62-64], while one study reported that SS carriers did in fact have fewer positive treatment gains [56], which would argue against a dissociation relative to pharmacological findings. Studies are required in which patients are randomly assigned to pharmacotherapy or CBT to fully test this hypothesis. One possibility is that interactions with environmental variables and differing etiological pathways into and out of mood and anxiety disorders may partly account for these differential findings.

To date, no studies have explored gene-environment interplay (e.g. with SLEs and early developmental experiences such as maltreatment) in predicting treatment outcome for psychological therapies. This would require large samples due to testing mutually dependent influences of multiple factors and accurate and in-depth characterization of environmental exposure history, ideally both negative and positive environmental exposures, treatment outcomes and the collection of genetic material [7]. Despite these methodological challenges, we believe that this would be a promising line of research, although it is unclear at this stage whether G×E interactions will have sufficient predictive value to guide personalized treatment decisions. Two recent studies identified that carriers of the SS genotype of the 5*HTTLPR* responded poorly to SSRI treatment but this association was only observed in those who experienced a stressful life event prior to treatment [142,143]. These findings are consistent with a combination of stress and greater genetic risk to stress predicting poorer response. However, this finding did not replicate in a third study [144]. While as yet untested and in contrast to findings from antidepressant studies, one possibility is that increased genetic vulnerability to stress (e.g. presence of the 5*HTTLPR* SS genotype) in combination with the presence of a stressful life event may in fact be associated with a better response to psychological therapies. In the antidepressant literature, significant G×E interactions have been reported between exposure to SLEs and SNPs in *FKBP5* (rs1360780) and *CRHR1* (rs110402) (see [18] for a review). However, here a combination of exposure to life stress and presence of the putative risk allele predicted a better treatment response. As suggested by others, disentangling the etiological pathways to mood and anxiety disorders may make it possible to differentially predict which individuals are at an increased likelihood of responding to pharmacological or psychotherapeutic interventions [18].

## Conclusions

Genes, environments, and gene-environment interactions contribute causally to mood and anxiety disorders. In this article, we have considered the evidence for genetic factors also being implicated in predicting individual variation in response to psychological treatment interventions. Therapygenetics, the study of genetic predictors of response to psychological therapy is a field very much in its infancy. Early findings have been somewhat mixed but several studies have provided promising evidence that individuals respond differently to psychological interventions and that genetic differences are capable of predicting differential susceptibility to psychotherapy. These findings show that interactions between genetic variation and environmental experiences (here, psychological therapy) may not only influence the development but also the remission of psychiatric outcomes. However, as yet these findings have not been independently replicated and the strength of findings are not sufficient to suggest immediate practical applications. We have suggested several methodological factors that warrant careful consideration in future research. We would strongly encourage clinical researchers to incorporate the collection of a genetic sample into their treatment protocols. Genetic variation can be measured with little error and remains stable over time and it is reasonable to hope that combining genetic information with clinical and environmental information or other biomarkers may prove to be particularly informative in guiding treatment selection, improving outcome and providing a better understanding of psychopathology.

## Competing interests

The authors declare that they have no competing interests.

## Authors’ contributions

KL undertook the literature searches and drafted the manuscript. TE helped plan the manuscript and revised a draft. All authors read and approved the final manuscript.
